# Recent Trends in Three-Dimensional Bioinks Based on Alginate for Biomedical Applications

**DOI:** 10.3390/ma13183980

**Published:** 2020-09-08

**Authors:** Farnoosh Pahlevanzadeh, Hamidreza Mokhtari, Hamid Reza Bakhsheshi-Rad, Rahmatollah Emadi, Mahshid Kharaziha, Ali Valiani, S. Ali Poursamar, Ahmad Fauzi Ismail, Seeram RamaKrishna, Filippo Berto

**Affiliations:** 1 Department of Materials Engineering, Isfahan University of Technology, Isfahan 84156-83111, Iran; farnoosh.pahlevanzadeh@gmail.com (F.P.); mokhtarirezahamid@gmail.com (H.M.); remadi@cc.iut.ac.ir (R.E.); ma.kharaziha@gmail.com (M.K.); 2 Department of Anatomical Science, School of Medicine, Isfahan University of Medical Sciences, Isfahan 81746-73461, Iran; valiani@med.mui.ac.ir; 3 Advanced Materials Research Center, Department of Materials Engineering, Najafabad Branch, Islamic Azad University, Najafabad, Iran; 4 Biomaterials, Nanotechnology, and Tissue Engineering Group, Advanced Medical Technology Department, Isfahan University of Medical Sciences, Isfahan 81746-73461, Iran; ali.poursamar@amt.mui.ac.ir; 5 Advanced Membrane Technology Research Center (AMTEC), Universiti Teknologi Malaysia, Skudai 81310, Johor Bahru, Johor, Malaysia; afauzi@utm.my; 6 Department of Mechanical Engineering, National University of Singapore, 9 Engineering Drive 1, Singapore 117576, Singapore; seeram@nus.edu.sg; 7 Department of Mechanical and Industrial Engineering, Norwegian University of Science and Technology, 7491 Trondheim, Norway

**Keywords:** 3D bioprinting, bioinks, alginate, natural polymers, cell-biomaterial interaction, biofabrication, regenerative medicine, tissue engineering, biomaterials

## Abstract

Three-dimensional (3D) bioprinting is an appealing and revolutionary manufacturing approach for the accurate placement of biologics, such as living cells and extracellular matrix (ECM) components, in the form of a 3D hierarchical structure to fabricate synthetic multicellular tissues. Many synthetic and natural polymers are applied as cell printing bioinks. One of them, alginate (Alg), is an inexpensive biomaterial that is among the most examined hydrogel materials intended for vascular, cartilage, and bone tissue printing. It has also been studied pertaining to the liver, kidney, and skin, due to its excellent cell response and flexible gelation preparation through divalent ions including calcium. Nevertheless, Alg hydrogels possess certain negative aspects, including weak mechanical characteristics, poor printability, poor structural stability, and poor cell attachment, which may restrict its usage along with the 3D printing approach to prepare artificial tissue. In this review paper, we prepare the accessible materials to be able to encourage and boost new Alg-based bioink formulations with superior characteristics for upcoming purposes in drug delivery systems. Moreover, the major outcomes are discussed, and the outstanding concerns regarding this area and the scope for upcoming examination are outlined.

## 1. Introduction

The body system has restricted functionality regarding regeneration. Recent treatment choices to substitute impaired tissue and organs depend on acquiring tissue through an identical person, or transplantation from cadavers that have been developed very quickly. However, there are restrictions to these treatments that consist of donor position, its side effect, and donor shortage [1,2]. These conditions additionally support the demand regarding biological replacements and the areas of tissue engineering (TE), and thus regenerative medicine is an effective route in the direction of regeneration development. Work in the area has developed to generate what we consider to be an innovative area—regenerative technological know-how, described as the affluence of innovative materials engineering, stem cell technology, and clinical translation concerning the reproduction of sophisticated tissues and body organ devices [1,2]. Numerous approaches are employed in this context; among them, the additive manufacturing (AM) has captivated a lot of consideration. AM or 3D printing is designed to incorporate living cells in 3D biomaterials. This innovative system enables the automated and reproducible generation of 3D well-designed living tissues through depositing layer-by-layer biocompatible materials (typically including biochemicals) with a high-accuracy placing of cells [1,2,3].

This approach enables the manufacturing of 3D, scalable and accurate geometries, which are generally not provided through other methods including 2D and 3D cell cultures [2,3,4]. Initially, Charles Hull created 3D printing [5], which he described as “stereolithography”, in the beginning of the 1980s, and from then on, this technology has developed into numerous kind methods [5]. All 3D printing techniques provide positive aspects and negatives [5,6]. The kind of 3D printer selected regarding an application typically relies on the components to be employed and precisely how the layers in the completed product are attached. The three most frequently used 3D printer technologies for medical purposes are selective laser sintering (SLS), thermal inkjet (TIJ) printing, and fused deposition modeling (FDM) [6,7]. Biomaterials employed in AM approaches comprising cells, base structure material, and some other necessary components are known as “bioinks”. The bioink particles are multicellular aggregates typically in the form of cylinders made up of cell types dependable with the tissue or organ system to be prepared [8]. A perfect bioink material must have a number of characteristics, including printability, great mechanical stability, insolubility in the physiological solution, suitable degradation rate that fits the aimed tissue, cytocompatible, and non-immunogenic [8,9]. Moreover, bioink materials ought to be created rapidly and scalable intended for industrial progress.

Hydrogels are suggested as appealing components for bioinks [9] due to the fact that they are biocompatible, present low cytotoxicity, and presence of a great amount of water provides them a structural likeness to ECM [10]. Typical hydrogels examined for 3D bioprinting are natural polymers, including chitosan (CS), and Alg [11]. Alg is an anionic polysaccharide attained through brown seaweed. The base-material taken out from seaweed is recognized as sodium Alg [12,13,14,15,16,17,18,19,20,21]. The phrases Alg and sodium Alg have generally employed alternately [22]. The Alg, among the widely recognized biopolymers, might entrap water and other molecules through making use of capillary forces and even now may permit it to penetrate from inside out. This specific feature is perfect regarding 3D bioprinting bioinks [2,23]. Alg bioinks need to have adequate viscoelasticity to accomplish injectability throughout the printing practice (viscosity features) and great pattern fidelity to sustain the created scaffold’s general shape, right after printing [9]. 

Considering that the viscosity of Alg bioinks relies on the Alg amount, the Mw of Alg, and the cell density encapsulation, printability is usually enhanced via manipulating these parameters [24]. An additional essential rheological characteristic of aqueous Alg solutions that experts have to consider is the shear-thinning, in which the viscosity reduces as the shear rate improves. The viscosity likewise relies on the temperature at which usually the printing was carried out; the viscosity diminishes as the temperature escalates [25]. Another examination presented Alg as an ideal polymer intended for printing [26,27,28]. In this review article, Alg is considered as a bioink, with its advantages and shortcomings. Additionally, its use in hard and soft tissues reproduction is reviewed, which could result in assisting experts for acquiring solutions toward Alg-based bioinks drawbacks, in order to accomplish an ideal combination and approach.

## 2. Alginate (Alg) as a Printable Material

**Figure 1:**
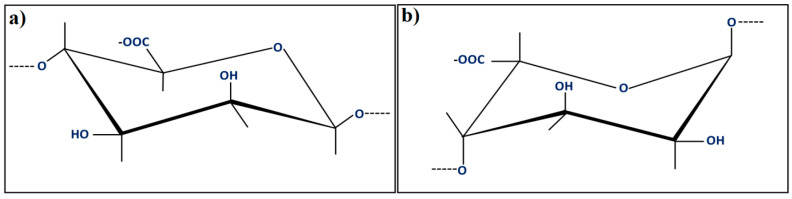
Units of the alginate block types: (**a**) β-(1-4)-d-mannuronic acid; (**b**) α-(1-4)-l-guluronic acid [2].

Alg is a natural biopolymer achieved through the processing of brownish algae, and is an adversely charged polysaccharide. Alg polymer includes a pair of monomers as a duplicating unit, which is (1-4)-β-d-mannuronic acid (M) and α-l-guluronic acid (G), as can be observed in Figure 1 [2]. However, α-l-guluronic acid stimulates the gel creation, the (1-4)-β-d-mannuronic acid, and a mixture of (L-4)-β-d-mannuronic acid and α-l-guluronic acid offers flexibility in the polymer matrix [29,30,31,32,33,34,35,36,37,38,39]. 

The molecular weight, shown as an average of all the molecules existing in the specimens, of Alg, varies around 33,000–400,000 g/mol. The 1% *w*/*v* aqueous Na-Alg solution possesses a dynamic viscosity 20–400 mPa⋅s at 20 ℃. Alternatively, Alg solubility is restricted through the solvent pH (a reduction in pH might result in polymer precipitation), ionic strength, and the number of gelling ions [40,41]. However, the cytocompatibility of Alg is substantially examined in vitro along with in vivo. There still exists an issue concerning the influence of the Alg composition. However, most of this misunderstanding probably pertains to numerous degrees of purity in the Alg examined in several studies. The immunogenic reaction at the injection or implantation sites could be caused by impurities keeping in the Alg. Considering that Alg is acquired through natural resources, several contaminants, including heavy metals and endotoxins, might exist. Significantly, Alg purified through a multi-phase extraction treatment to an extremely high purity did not stimulate any substantial foreign body response as soon as implanted directly into animals [42]. Alg would not induce considerable inflammatory reaction when applied in an in-vivo condition, consisting of the use of bioinks for 3D-printers. Injectable Alg bioinks are among the most usable and practical materials for bioprinting on account of their shear-thinning capacity, quick crosslinking, and possibility of cell printing [24]. By combining with solutions comprising multivalent cations, such as LiCl, BaCl_2_, and so on, Alg is ionically crosslinked to create a 3D hydrogel, presenting an ECM mimicking atmosphere for cells, in addition to outstanding printability, structural stability, and mechanical strength [42]. 

Pores sizes in Alg vary around 5–200 nm, and the most oversized pores are observed in great-G-block-amount Algs. This kind of character is essential concerning the biocompatibility of the bioink (as a result of a restricted infiltration of nutrients). Additionally, the concentration of an Alg-based bioink varies according to the Alg amount, the Mw of the Alg employed, and the cells’ characteristics. These are the factors that investigators need to consider to be able to adjust the concentration of the Alg-based bioinks [2]. Through modifying G/M ratio in Alg structure, weight ratio, solid amount, along with cell amount, the viscosity, mechanical and structural characteristics of the Alg based bioinks might be adjusted to match the necessities of various bioprinting strategies and various biomedical fields. Even so, several undesirable features, for instance, poor long-term structural stability and mechanical characteristic, uncontrollable degradation rate, and the bioinert properties, ought to be regarded about Alg-based bioinks for bioprinting purposes [42,43,44]. 

## 3. Applications in Biomedical Field

**Figure 2:**
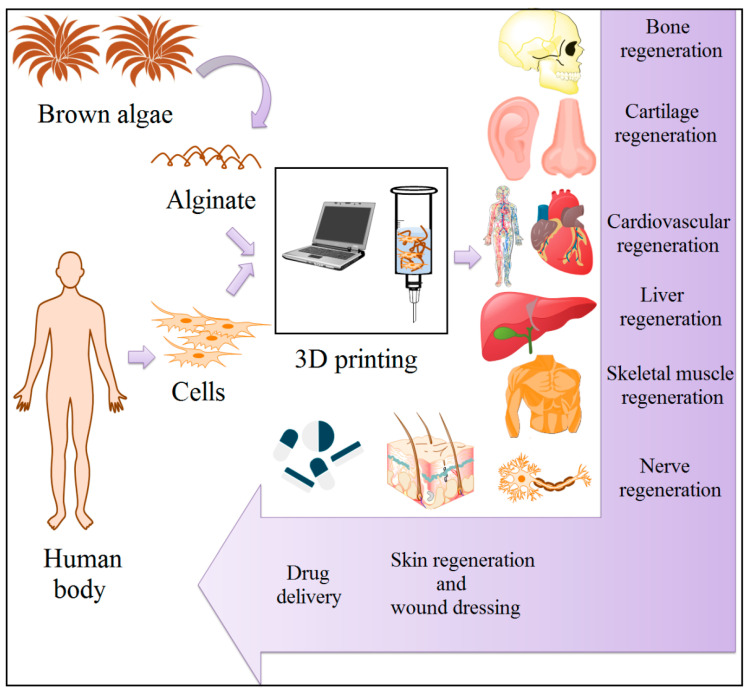
Various application of 3D printed alginate constructs in tissue engineering.

Alg constructs created through various approaches have been looked into for the numerous tissues reproduction [44,45,46,47,48,49,50,51,52,53,54,55,56,57,58,59,60,61,62,63,64,65,66,67,68], delivery of a wide range of low molecular weight (Mw) drugs [69,70,71,72,73,74,75,76,77,78,79], wound dressing [80,81,82,83,84,85,86] and cell loading [87,88,89]. Furthermore, Alg’s structural and mechanical features needed for printing of each one tissue, along with the biomimicry characteristics required in every single case, might be adjusted by either encapsulation of other kinds of biomaterials in Alg-based matrix or by utilizing various hydrogel manufacturing methods. There is currently a commercially accessible bioink called CELLINK, which usually is a combination of nano-cellulose and Alg. CELLINK provides shear-thinning capacity and rapid crosslinking characteristics, rendering it useful regarding soft TE fields [90]. Moreover, incorporating synthetic polymers and bioceramics, including PCL and PLA into Alg-based inks, might cause them to become appropriate in hard TE fields [91,92]. These endeavors are demonstrated in Figure 2 and explained in the following sections in detail. In this context, various biomedical fields of Alg are exhibited in Table 1 [44,45,46,47,48,49,50,51,52,53,54,55,56,57,58,59,60,61,62,63,64,65,66,67,68,69,70,71,72,73,74,75,76,77,78,79,80,81,82,83,84,85,86,87,88,89].

Alg: alginate; AAlg: aminated alginate; AlgDA: alginate dialdehyde; AlgGO: alginate gel bead containing vegetable oil; BMCs: bone marrow cells; BSA: bovine serum albumin; BT: bone tissue; CAlg: calcium alginate; CaGlu: calcium gluconate; CMC: carboxymethyl cellulose; CPC: calcium phosphate cement; CS: chitosan; CTE: cardiac tissue engineering; CTTE: cartilage tissue engineering; GL: gelatin; GRGDSP: glycine–arginine–glycine–aspartic acid–serine–proline; HAp: hydroxyapatite; hBMSCs: human bone mesenchymal stem cells; HDF: primary human dermal fibroblast cells; HOS: human osteosarcoma cell lines; HNTs: halloysite nanotube; HPMC: hydroxypropyl-methylcellulose; hUCMSCs: umbilical cord mesenchymal stem cells; LVM: high M content; MSCs: mesenchymal stem cells; MVG: high G content; MZ: metronidazole; Na-Alg: sodium alginate; NHDF: normal human dermal fibroblasts; NPCs: neural progenitor cells; PC: pectin; PEO: poly(ethylene oxide); PLGA: poly(lactide-co-glycolide; PNIPAAm: poly(N-isopropylacrylamide); PVA: poly(vinyl alcohol); PVP: polyvinyl pyrrolidone; SIM: simvastatin; 5-FU: 5-fluorouracil; TE: tissue engineering; TGF-1: transforming growth factor 1; UV light: ultraviolet light; BMP-2: bone morphogenetic protein 2; BTE: bone tissue engineering.

### 3.1. Bone Regeneration

Bone defects with small size might be fixed through the self-healing performance of bone tissue (BT), however massive bone defects might merely be fixed via BT transplantation. The current examination methods for restoring impaired bone tissue are bone tissue engineering (BTE) methods, because of the issues encountered in acquiring materials regarding an autogenous bone graft [93,94]. In this investigation, 3D bioprinting approaches were utilized as an appealing approach in the current years [95,96]. Compared to conventional BTE, 3D bioprinting has superb possibilities to create tissues with numerous biomaterials, cells, and bioactive materials in a patient, which are certain disorder forms [97]. Nevertheless, BT bioprinting requires inks with appropriate viscosity, mechanical performance, and apatite formation capacity to enhance bioactivity and create chemical bonds with adjacent BT following implantation [98,99].

**Figure 3:**
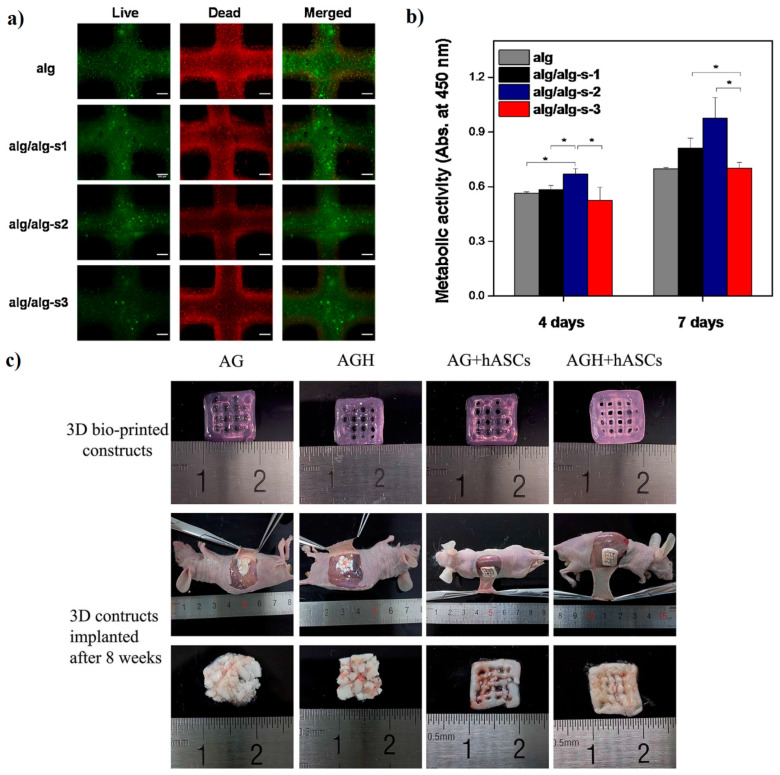
Osteoblasts viability and metabolic activity in 3D printed Al/Al-sulfate bio-inks: (**a**) fluorescence images of live/dead; (**b**) 3D-printed cells metabolic activities in hydrogels at day 7 of incubation [102]; (**c**) morphological analysis before and after implantation [98].

One essential factor in employing hydrogels in BTE is their mineralization capacity [100]. Detsch et al. [101] revealed that mineralization of hydrogels, attractive for bone tissue (BT) regeneration purposes, might be accomplished enzymatically through encapsulation with alkaline phosphatase (ALP). A 3D BioPlotter was employed to create Alg scaffolds with the aim of improving cell viability and ALP. They observed the mineralization in the entire scaffolds encapsulated with ALP, which might enhance the mechanical performance. Therefore, it appears that the incorporation of enzymes such as ALP into hydrogels might cause them to become suitable substrates for BTE, comprising the potential of attachment to the natural tissues [101]. The effect of different amounts of Alg-sulfate from 5 to 30 mg/mL on the characteristic of Alg inks was evaluated by Park et al. [102]. Cell printing results disclosed that cell viability and osteogenesis level enhanced in the hydrogels loaded with Alg-sulfate than that of the control bioinks. In this context, the 3D-printed cells encapsulated into different bioinks exhibited a great cell viability Figure 3a and metabolic activity (Figure 3b) [102]. Typically, between bioinks, Alg/Alg-s2 (1–3 wt.% Alg-s) was introduced as the most appropriate formulation for enhancement of bone regeneration regarding cell growth and Ca-precipitation.

One of the desirable approaches for producing Alg-based inks is encapsulated with bioceramics into a polymeric matrix. An in vivo experiment was conducted by Wang et al. [98] regarding printed sodium Alg/Gel/hASCs (AG construct) and sodium Alg/Gel/nHAp/hASCs (AGH construct) and attained constructs implanted in mice for eight weeks. To evaluate bone formation ability, micro-CT scans were carried out. Outcomes pointed out that the no-cells-encapsulated AG construct and no-cells-encapsulated AGH construct had been reasonably loose. Furthermore, the hydrogel-based constructs pointed out the soft and brittle structure that made the constructs hard to maintain the following implantation. The AG and AGH construct encapsulated with hASCs cells kept the initial shape, with minor alterations in appearance; the structure was reasonably tough, and cell growth was noticeable via the pores (Figure 3c) [98]. Furthermore, osteogenic differentiation of AG and AGH construct encapsulated with hASCs cells exhibited that the presence of nHAp causes enhancement of osteogenic differentiation of the AGH constructs in vitro and in vivo experiments, which making it suitable for BTE applications [98].

In another study, Egorov et al. [27] encapsulated calcium phosphate (CP) into sodium Alg construct employing a fairly basic method. The solid connection among the –COOH group of Alg and the HPO_4_^2−^ supplied this gel’s integrity and uniformity. They likewise exhibited the compressive strength of construct escalated with Alg amount from 0.45 to 1.0 MPa [27]. In other research conducted by Luo et al. [26] Alg/Gel scaffolds fabricated via 3D printing coated with nano-apatite. Their finding depicted that the coated scaffolds presented outstanding mechanical performance and appealing bioactivity, turning it into desirable as an excellent scaffold for BTE, scaffold Young’s Modulus enhanced twice compared to the uncoated scaffold, even though stem cells proliferation enhanced [26]. Bioactive glass (BG) is another kind of desirable incorporate agent to improve bioactivity and mechanical characteristics of Alg based constructs. In this case, composite scaffolds containing 13–93 wt % BG and sodium Alg (BG/SA), were fabricated for BTE using 3D printing approaches via Luo et al. [28].

Their result depicted that encapsulation of low amount BG (33.3% wt %) noticeably enhanced the compressive strength and its modulus than that of SA scaffold without BG. Besides, in vitro apatite formation, cell attachment, and osteogenic differentiation escalated owing to the release of bioactive ions, including Mg^2+^ and SiO_4_, from scaffolds encapsulated with BG. Another report revealed that carbon-based nanomaterials, as a result of their appealing mechanical features, were employed as an incorporate agent in Alg-based bioinks for BTE. Choe’s et al. [103] showed that mechanical performance and cell response improved after embedding GO (0.05–1.0 mg∙mL^−1^) into Alg-based bioinks [103]. Their study also points out that the addition of 0.5 mg∙mL^−1^ GO as an optimum amount into Alg bioink leads to enhancement of printability, structural integrity, and cell interaction [103].

Besides ceramic-based materials, the encapsulation of peptides is an additional appealing strategy to enhance Alg bioink characteristics for BTE. Heo et al. [104] encapsulated bone formation peptide-1 (BFP1) into the Alg-based construct. Both in vitro and in vivo examination depicted that the Alg-based construct offered a stable atmosphere for the hADSCs response, which resulted in bone reproduction enhancement [104]. Their results point out that bone defects could be reconstructed via anti-inflammatory agents. Likewise, Wang et al. [105] prepared 3D-printed Alg/nHAp scaffolds containing Atsttrin with the aim of accelerating the bone healing rate. Their results revealed that a 3D-printed scaffold is able to release Atsttrin for 60 h and reduce the inhibitory influence of tumor necrosis factor-α (TNF-α) on BMP-2 bone morphogenetic protein 2)-induced osteoblastic differentiation. Typically, their results showed that the accurate design and anti-inflammatory response of the scaffolds containing Atsttrin could facilitate bone defect restoration [105]. Taken together, blending Alg with other polymers and incorporation of additive agents including nHAp, BG, GO into the blend polymer containing Alg could enhance mechanical characteristics of 3D printed scaffolds for BTE applications.

### 3.2. Cartilage Regeneration

**Figure 4:**
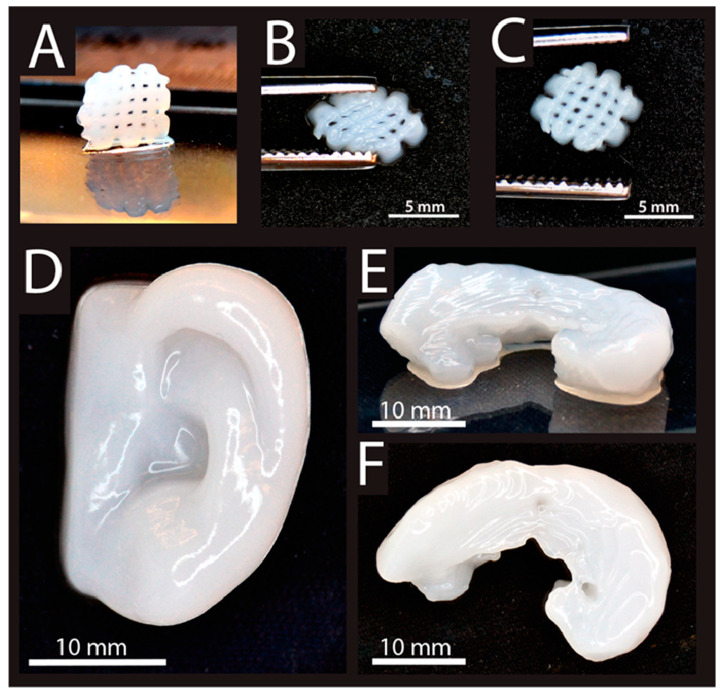
(**A**) 3D printed grids (7.2 × 7.2 mm^2^). (**B**) The deformity of grids with squeezing, and (**C**) restoring after squeezing. (**D**) 3D printed human ear; (**E**) (side view) and (**F**) (top view)) [107].

Articular cartilage disorders have restricted efficiency for self-reproduction and recovery. Cartilage injuries frequently lead to discomfort and reduced performance for the affected person and usually hasten the progress of osteoarthritis in the joint. 3D bioprinting might provide treatment solution options which may possibly conquer the restrictions of current management options, including the creation of fibrocartilage, donor site morbidity, hypertrophy of implant. The mixture of cells, natural-based materials, and biochemical components could present the opportunity of true cartilage reproduction [106]. In this perspective, Alg is extensively used in cartilage 3D bioprinting. Markstedt et al. [107] evaluated a shear thinning bioink of NFC encapsulated Alg with the rapid cross-linking capacity regarding the 3D bioprinting of TE. Their 3D product with a small cross-linked grid working as a rigid gel is shown in Figure 4A–C [107]. In Markstedt et al. [107] investigated the shapes similar to cartilage tissues, including an ear and a meniscus had been effectively printed, Figure 4D–F. Actually, with printing periods of as much as 20 min for these larger sized constructs, the printed products lose their form throughout the printing procedure because of the ink’s viscosity. It appears that their ink seemed to be among the appealing materials regarding cartilage tissue printing in complicated shapes in the appropriate manufacture period [107].

Muller et al. [108] fabricated Alg sulfate-based bioink loaded with nanocellulose. They revealed that when the bioink was basically printed, the biological response of the cells was extremely influenced by the nozzle dimensions and shape. Cell proliferation and growth were preserved along with the lowest extrusion pressure and shear stress. Nevertheless, extruding the Alg sulfate/nanocellulose bioink and chondrocytes considerably affected cell viability, especially if employing nozzles and valves with a small diameter. For this reason, the choice of needle shape and bioink requires tuning the factors for great printing quality and great cell viability, in addition to cell growth, structure and matrix deposition [108].

In another study performed by Yang et al. [109], the mixture of sodium Alg (SA) based bioinks with other natural polymers (collagen type I (COL) or agarose (AG)) for cartilage reproduction was investigated. Their outcomes demonstrated that the mechanical characteristic of Alg bioink was enhanced in both SA/COL and SA/AG groups than that of neat SA. Nevertheless, 3D bioprinted SA/COL as a suitable printed sample with more appropriate mechanical strength and biological response successfully diminished the dedifferentiation of chondrocytes and maintained the phenotype [109]. Accordingly, Kundu et al. [110] encapsulated PCL into Alg bioink-based (PCL–Alg scaffolds) concerning cartilage TE via AM coupled with multihead deposition methods. According to their finding, cartilage tissue and collagen fibril formation improved based on the in vivo study [110]. Ultimately, their conclusion exhibited that Alg as a biostable hydrogel with a sluggish degradation rate and suitable mechanical characteristics that might be an appropriate option for cartilage bioprinting.

### 3.3. Cardiovascular System Formation

Heart breakdown is an escalating issue that is very prevalent in elderly Western inhabitants, with an occurrence in more than 20 million people throughout the world. Present heart problem treatments are incapable of turning back heart failure and usually do not tackle its primary cause, the impairment of cardiomyocytes [111]. The emerging field of TE and drug delivery system retains outstanding guarantees as a vital strategy regarding generating tissues to restore congenital issues and impaired cardiac tissues. Among the innovative manufacturing approaches, 3D printing uses automated operations and consistent components as creating blocks and makes it possible for the generation of 3D items from customized computer-assisted designs. The AM strategy (3D printing) was presented into TE fields by employing biopolymers for creating complicated and composite scaffolds [112]. In this perspective, Alg based hydrogels are among the appropriate materials in cardiac fields.

The more recent development in cardiac implementing Alg has currently resulted in novel standards in biomaterial fields in the cardiac system to not merely “fill the gap” in the damaged region, but to behave as an interface with the cardiac biological systems at the same time [113]. These kinds of applications concentrate on several main areas: (1) employing Alg hydrogels such as an ECM substitute in heart tissues to enhance tissue reproduction because of the structural likeness concerning Alg and organic heart ECM, (2) employing Alg hydrogels for delivery system regarding cardiac stem cells or mature cardiomyocytes to the damage region to enhance the reproduction of practical heart tissue, (3) employing Alg for a platform regarding the constant release of growth factors to simulate organic physiology, and (4) applying Alg gels to handle drug delivery. As a drug delivery system, an Alg-based biopolymer might be great-tuned to handle the velocity of cardiac drugs released by managing the cross-linker kind and technique [114]. From this perspective, Duan et al. [115] applied 3D bioprinting to prepare living Alg/Gel hydrogel valves, and their findings revealed that cells were alive inside Alg/Gel hydrogel construct for seven days. Similarly, their finding depicted that complicated, non-uniform, and loaded aortic valve hydrogel might be prepared using 3D printing [115].

**Figure 5:**
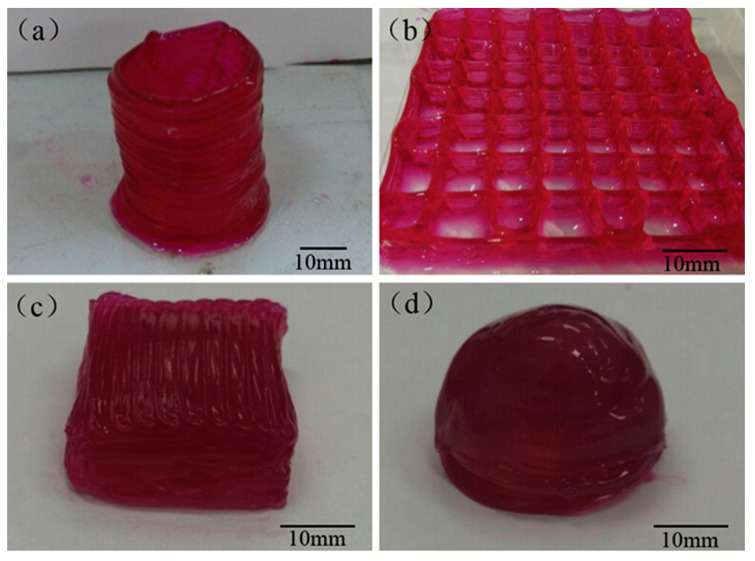
3D printed Alg structures with built-in microchannels: (**a**) hollow cylinder, (**b**) grid, (**c**) cuboid, and (**d**) hemispheroid [118].

However, as separated areas that are lower than 3 mm^3^ [116], the printed components’ restricted vascularity is a serious obstacle regarding 3D printing of organ constructs [117]. The design of the blood vessel-like routes has the carrying ability, e.g., of oxygen and nutrients, by means of the printed material that is needed to be able to prepare huge tissues and organ constructs. In this context, Gao et al. [118] synthesized Alg-based constructs containing microchannels encapsulated with sodium Alg and calcium chloride solutions. They printed numerous 3D constructs containing microchannels in the form of a hollow cylinder (Figure 5a), a grid (Figure 5b), a cuboid (Figure 5c), and a hemispheroid (Figure 5d) to illustrate the functionality of this approach [118]. Hence, the approach might be applied to prepare several kinds of constructs containing these microchannels for presenting tissue and organ constructs.

Numerous studies regarding 3D bioprinting of Alg-based constructs were conducted pertaining to vascular application. For example, Zhang et al. study [86], embedded HUVSMCs cells into Alg, and subsequently, CaCl_2_ solutions were furnished via the shell and core areas of the nozzle. By starting the process, crosslinking initiated quickly and thus creating a canal. Perfusable vasculature canals with specific sizes were printed. The suggested bioprinting procedure was suitable for preparing a vasculature canal of different lengths within a short manufacturing period and might be effortlessly encapsulated into prepared thick tissue, and 3D printed organs. Zhang et al.s’ [119] study likewise illustrated various positive aspects such as no requirement for post-manufacturing treatment and permitting direct bioprinting of complicated shape along with a well-designed branched system. There is another examination [120], which employed sodium Alg to create bioink for cellular tube preparation. In this context, their findings verified that the bioink is composed of 3T3 cells, sodium Alg and cell medium, which can aid 3D bioprinted blood vessels with complicated shapes. Likewise, Christensen et al. [121] used sodium Alg and mouse fibroblast–based Alg bioinks to print a vascular-like system. Another study [122], printed a perfusable vascular system via mixing sodium Alg with gelatin methacryloyl (GelMA) and 4-arm poly(ethylene glycol)-tetra-acrylate (PEGTA). Taken together, this concludes that Alg-based bioinks are one the most important natural polymers to 3D bioprint vascular tissue and blood vessels via coaxial needle systems, owing to the quick ionic crosslinking capacity of the Alg. Moreover, Alg-based bioinks presented excellent gelation kinetics along with reasonably high accuracy by modifying the amounts of the Alg and the crosslinker.

### 3.4. Regeneration of Other Tissues

Alginate, which is typically employed in 3D bioprinting of numerous tissues, offers low toxicity and enhanced biocompatibility and is relatively inexpensive compared to some other biomaterials [123]. Using Alg gels as a bioink also being actively investigated for their ability to intercede the regeneration of various other tissues and organs, including skeletal muscle, nerve, skin, and liver. Transplantation of pancreatic islets might reduce unstable blood-glucose control in some patients with diabetes type 1. Defense of these islets through the immune system might be achieved by incorporating a hydrogel, the majority of which is Alg. As an illustration in a vital investigation by Duin et al. [124], islet incorporation coupled with 3D extrusion bioprinting employs Alg and methylcellulose, making it possible for loading pancreatic islets in 3D hydrogel constructs of described geometry though maintaining their viability, structure, and performance. The loaded islets regularly generate insulin and glucagon in the course of the treatment [124].

In another study, Chang et al. [125], develop a viable direct cell writing process for biofabrication of reproducible three-dimensional cell-encapsulated Alg-based tissue-engineered constructs within three-dimensional tissue chambers for liver as a drug metabolism model. After printing properly, they achieved high-throughput reproducible biofabrication of tissue constructs with precise patterning, direct integration with the microfluidic platform. The printed structure enhances cell viability and controls cellular-level differentiation and tissue-level function. The technology presented in their work was a structurally and biologically viable syringe-based direct cell-writing process for layer-by-layer fabrication of three-dimensional cell-encapsulated hydrogel-based tissue constructs [125,126]. In a study, Ning et al. [127] used Alg-based biomaterials for developing 3D cell-laden constructs. Schwann cells were studied, and the temperature and bioink concentration were observed to investigate the effect of flow behaviors, during printing [91,92]. The influence of the mechanical properties of Alg-hyaluronic acid hydrogel on cell performance was evaluated, and a suitable range of stiffness was suggested to be used in tissue repair scaffolds for peripheral nervous system regeneration with living Schwann cells [126,127]. They offered the construct for the potential applications in nerve tissue regeneration as a treatment for trauma in peripheral nerves.

**Figure 6:**
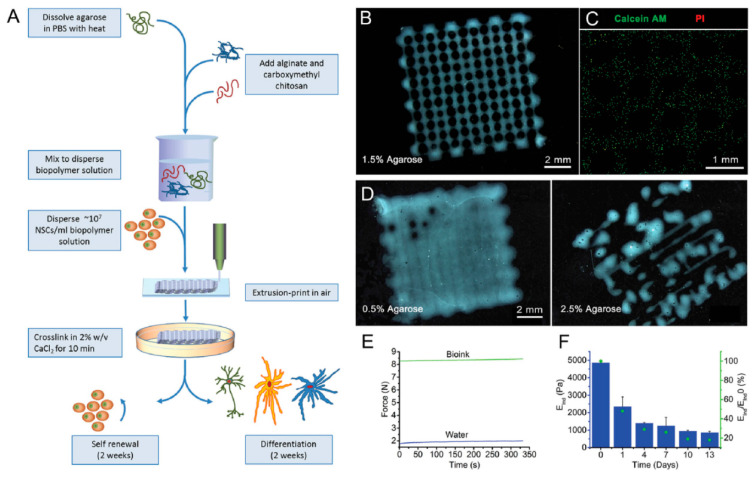
Production of hNSC-laden Al-CMC-Ag bioink. (**A**) Schematic presentation of hNSCs with bioink printing for 3D cell culture and differentiation. (**B**) Printed gel constructs containing 5% *w*/*v* Al, 5% *w*/*v* CMC, and 1.5% *w*/*v* agarose. (**C**) Staining of Live and dead hNSC. (**D**) scaffold structures containing 0.5% and 2.5% *w*/*v* agarose (**E**) Optimal bioink (green line) demonstrated by force required for printing. Water used for comparison (blue line) and (**F**) optimal gel indentation modulus (blue bars) and % modulus (green dots) at a determined time relative to the primary modulus at day 0 [128].

Gu et al. [128] reported an Alg–agarose–carboxymethyl–chitosan blend to produce 3D printed structures with induced human-derived neural cells for developing functional neurons as shown in Figure 6. By the successful printing and formation of stable 3D structures with cells encapsulated in construct, the bioink represented as a potential, widely available, inexpensive, which formed a printable, clinically compatible gel [128]. Mozetic et al. [129] used a blend of Alg and Pluronic for investigating its effects on myoblast cell viability and alignment. Their study’s advantage is the possibility to produce many small muscle bundles in which myotubes are already aligned along the fiber direction, allowing tissue microarchitecture to be tailored during printing. Additionally, the advanced mechanical properties provided by Pluronic/Alg blend enable to overcome the extensive in vitro culture. Further gene expression confirmed the improved viability compared to the normal 2D cultures [129].

**Figure 7:**
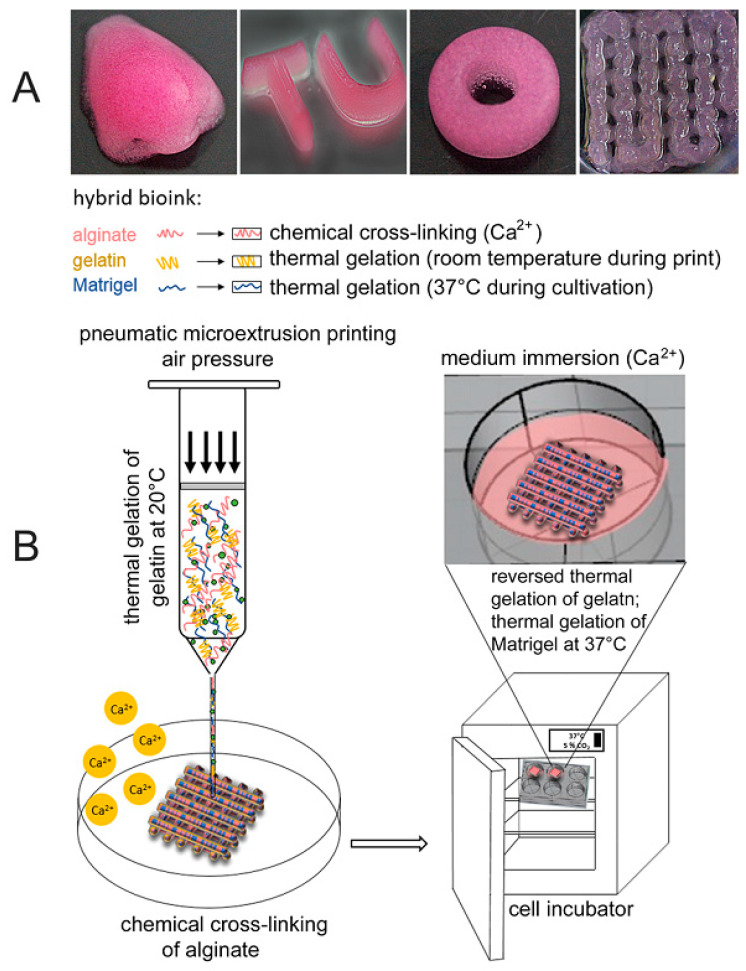
(**A**) Structures with different shapes produced by printing with the bioink consisting of 2% Alg, 3% Gel, and 20% Matrigel. (**B**) 3D printing procedure schematic [130].

In another study, Berg et al. [130] represent hydrogels consisting of Alg, gelatin, and Matrigel, which were used to provide a scaffold for a 3D arrangement of human alveolar A549 cells. Additionally, they studied infection of the 3D model with a seasonal influenza A strain resulting in widespread distribution of the virus that is also examined in the natural lung. By comparing the 3D structure with two-dimensional cell culture, they show the benefit of 3D printed constructs compared to conventional culture conditions. Viability and cell distribution were optimized by adjusting the Matrigel content in the printed bioink, which helps to print 3D lung models with A549 cells with better conditions. Due to the viral replication and proinflammatory interferon release of the infected cells in the bioink, they demonstrated that their procedure of bioprinting is a suitable way for the generation of humanized 3D tissue models, as can be seen in Figure 7 [130].

**Figure 8:**
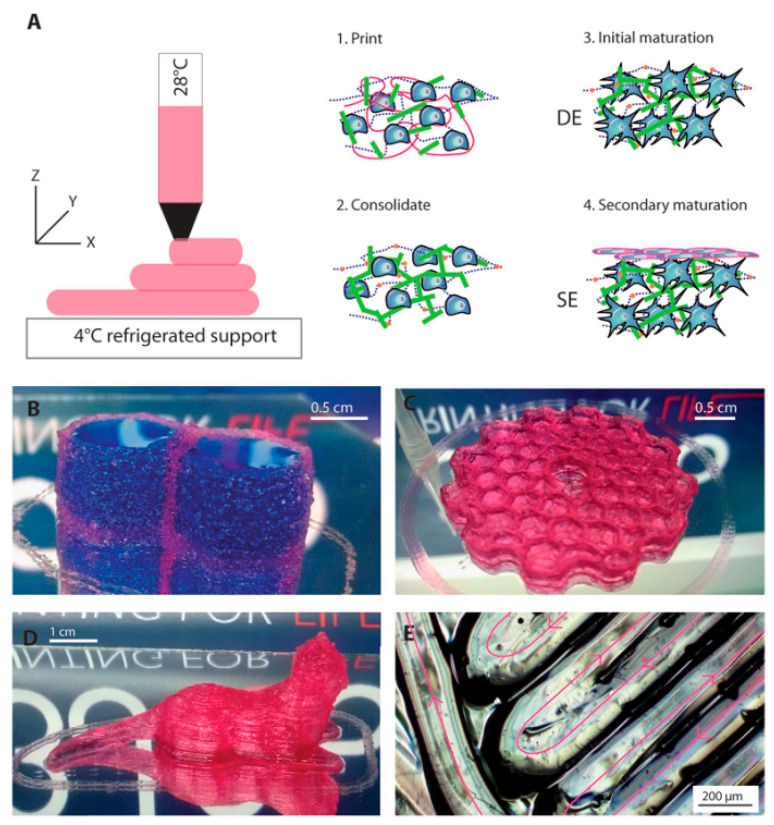
(**A**) Schematic representation of the 3D bioprinting, consolidation, and maturation steps. Examples of 3D printed constructs. (**B**) A water tight constructs with two compartments containing blue dye. (**C**) Honeycomb structure. (**D**) A centimeter size complex structure. (**E**) A closer view of printing [131].

Pourchet et al. [131] developed an Alg-based ink formulation and bioprinting process to produce a full-thickness skin engineered with primary human skin cells (Figure 8). The bioink is suitable for the fabrication of highly complex objects, and based on this fact, they can fabricate scaffold by printing the adult ear shape [131]. Additionally, they observed cells spreading in a 3D environment, which induced a rapid differentiation of the dermis, leading to a stiff tissue on which epidermis can be rapidly seeded. Through immunostaining and electronic microscopy, they demonstrated that the bio-printed skin presents all characteristics of human skin, both at the molecular and macromolecular levels. Finally, the printability of large skin objects is demonstrated with the printing of an adult-size ear. Their results offer significant advantages compared to the contemporary skin tissue engineering methods, by precisely positioning the biological and biochemical materials and living cells with spatial control [131].

**Figure 9:**
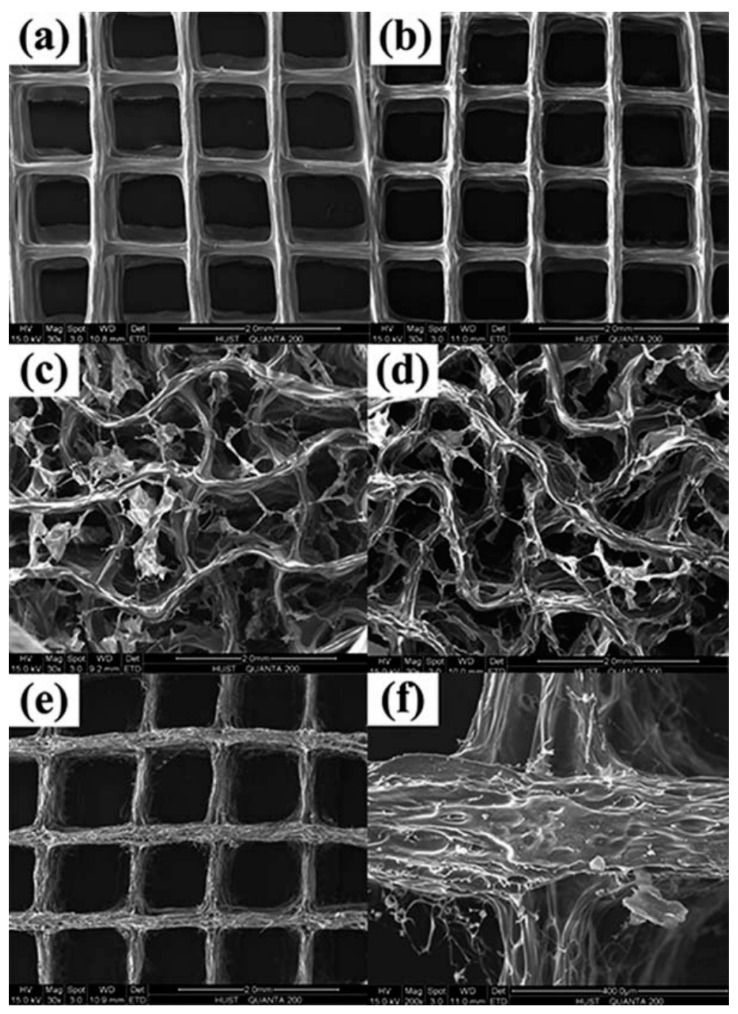
SEM images of different crosslinking groups. (**a**–**d**) Groups crosslinked with CaCl_2_, CaCl_2_–EDC, EDC, and EDC–CaCl_2_, respectively. (**e**) Blank specimen without crosslinking. (**f**) Blank group (enlarged view) [132].

Shi et al. [132] proposed a method to make Alg/gelatin scaffold for dermal substitute by bioprinting. They conducted different characterizations to study the influence of ionic (CaCl_2_), chemical (EDC), and dual (CaCl_2_-EDC) crosslinking processes (Figure 9). Experimental results showed that a dual crosslinking process could make dermal substitute scaffolds with physicochemical properties that match with human skin tissue and satisfy requirements of skin tissue engineering. They also do cell culture tests on human skin fibroblasts (HSFs), and the cell proliferation result also approves the scaffold’s biocompatibility. This research offers a new potential approach to make bioactive dermal substitute scaffolds [132]. In conclusion, suitable properties, such as biocompatibility, efficient crosslinking, tunable mechanical and rheological properties, and ability to blend formation with different polymers make Alg a famous bioink for 3D bioprinting different tissue and organs.

## 4. Wound Dressing

Skin is a protective barrier for the body, which is a complex structure containing pigmentation, vessels, hair follicles, and different cell types [133,134]. As a significant problem, wounds can put the skin’s health in great danger. Wound healing has many challenges, such as the presence of underlying illness, a high volume of exudates, microbial infection, less perfusion, and poor nutrition [134]. The currently clinical dressing strategies can generally be classified into (i) traditional dressings (e.g., gauze), (ii) skin graft (e.g., allograft, autograft, autologous split-thickness skin graft (ASSG)), and (iii) advanced dressings (e.g., medicated modern dressings and tissue-engineered substitutes) [135,136]. The efficiency of traditional dressing is limited; even with great ability to absorb and reasonable price, they can cause secondary injury, damage the newly formed epithelium and bleeding on removal, exhibit poor vapor permeation, and cause bacterial infections because of the leakage of exudates from these dressings [137,138]. Skin graft use is limited by various reasons, such as rejection by the body because of immune reaction, risk of infection and transmission of diseases, and the number and size of donor sites, which leads to further trauma, potentially resulting in additional complications [135,137].

The limitations mentioned earlier have prompted the advance of skin tissue engineering. Compared to other tissue engineering technologies in advanced dressing, 3D bio-printing technology has been considered as a promising strategy in recent years [135]. 3D bio-printing provides a unique ability to assemble biomaterials and cells to build 3D structures of the skin. By using computer-aided design software, it allows more flexibility and repeatability since 3D structures can be designed based on predetermined sizes and porosities [133]. These fine porosities can lead to better accessibility by body fluids, and cells resulted in better regeneration and mimicking the natural organization of healthy skin. In addition, fabrication of skin replacements with embedded ECM components, growth factors, and cell types based on each region and wound depth, facilitating the body’s natural response to the trauma while protecting the wound site [139]. For bio-printing, essential factors involve bioink formulation, which affects the printing process, and consequently, the biological and mechanical features of the result. For instance, fabrication of interpenetrating polymer network (IPN) or nanocomposite hydrogel, results in changing shear-thinning behavior, which can be tailored to mimic the final tissue behavior.

Recently, Alg and linear block copolymer represent the high biocompatibility due to the support of epidermal cell growth, which makes it a suitable candidate in wound dressing [137]. Thanks to the shear-thinning property, the Alg solution is an ideal bioink for 3D bio-printed tissue-engineered constructs. Liu et al. [140] fabricated the gelatin-Alg scaffold by 3D bioprinting. They used the scaffold for skin wound healing on a mouse back and investigated the histopathological changes during the wound healing process (Figure 9). They tried to cover the wounds by the bioactive scaffold, including a layered gelatin-Alg polymer with regular and suitable size holes, which was printed. The results revealed the scaffold shortened the average wound healing. The average healing time of the control mice was 16 ± 1 days, while the same data for treatment mice by the gelatin-Alg scaffold was 14 ± 1 days as shown in Figure 9 [140]. Further histological analysis also revealed improved healing in the treatment of mice. The scaffolds reduced necrosis, hemorrhage, and inflammatory exudation to form thinner crusts than traditional dressing, at the early and middle stage [140].

The formation of granulated tissue with uniform thickness, the maturation of new capillaries, and declined to swell, all cultivated by gelatin-Alg scaffolds. It was observed that the scaffolds enhanced the detachment of the crusts, decreased the agglomeration of keratinized substances (Figure 9). As a result, the regeneration of squamous epithelium of uniform thickness and formation of dense collagen fibers facilitated, and thus, improved the strength and tension resistance of scar tissue [140]. In a similar study, Wang et al. [141] designed and printed a bilayer membrane (BLM) scaffold consisting of an outer poly (lactic-co-glycolic acid) (PLGA) membrane and a lower Alg hydrogel layer, which mimicked the skin epidermis and dermis, respectively. The first layer of PLGA nanofiber membrane was prepared using high voltage printing. In contrast, the second layer was fabricated by printing Alg hydrogel on the surface of the PLGA nanofiber membrane [141]. They observed porous Alg hydrogel promoted cell adhesion and proliferation, compared with PLGA. In contrast, the BLM scaffold with a PLGA layer was able to prevent bacterial invasion and maintained the moisture content of the underlying hydrogel. After implantation in the dorsal wound of rats, the PLGA and Alg hydrogel scaffold presented the ability to promote inflammation, neovascularization, and collagen I/III deposition and improved wound healing. They believed that the 3D-printed scaffold is an extraordinarily promising type of wound dressing and skin substitute [141]. Overall, Alg-based dressings is a proper choice in wound dressings, as it has been considered by high water uptake, increased porosity, and non-immunogenic effects. These properties enhance rapid re-epithelialization, granulation tissue formation, and wound healing [137]. Skin bioprinting is the fact that changes the future of wound dressing. This field probably needs both suitable materials and high-tech fabrication methods if it means to be advanced rapidly. Considering the tunable properties of Alg and the ability to adapt to high-tech fabrication methods, this polymer seems to have an important role to play in the future of wound dressing.

## 5. Drug Delivery

Drug delivery refers to systems that act as a platform for the medicine to be delivered to specific parts of the body to apply its maximum therapeutic effect. Recently, drug delivery has expanded significantly with a current focus on targeted delivery to improve drugs’ efficacy and safety profile [142,143]. Researchers are looking for a highly personalized pharmaceutical treatment through tailored engineering release [144]. Alg is among the appropriate materials for drug delivery applications. As an illustration, extremely porous Alg scaffolds created with covalent crosslinking were applied to enhance biocompatibility in Wang et al. [145] study. Furthermore, a critical investigation employing Alg by Veiseh et al. [146], indicated that the in vivo cell viability of biomedical devices might be considerably increased by adjusting their spherical morphology. They exhibited that for incorporated rat pancreatic islet cells transplanted into diabetic mice were allowed to re-establish blood glucose control as much as 180 days, this duration five times greater than transplanted grafts incorporated inside traditionally sized 0.5 mm Alg tablets [146].

One of the crucial technologies that significantly improved pharmaceutical manufacturing is 3D printing. It is capable of developing a range of drug delivery systems that covers oral controlled-release systems, microchips, and drug implants to multiphase-release dosage forms [147]. This technology is able to design both simple and complex customized drug delivery systems [147,148]. 3D bioprinting is also one of the important techniques that use bioink to act as a medium to the tailored release of the drug. Between polymers, Alg has been widely used as pharmaceutical additives, such as a tablet disintegrate and gelling agent. The in situ gelling Alg system is an excellent drug carrier for the prolonged delivery of drugs [149]. Alg used in the form of hydrogel, matrices, membranes, films, microspheres, and beads in drug delivery. The bio-adhesive character with good viscoelastic behavior of Alg makes it useful in the pharmaceutical industry as a bioink [149]. One crucial technique in 3D printing that always attracts researchers is coaxial printers. Exciting research, Do et al. [150] designed a 3D structure for a controlled drug or molecule delivery system capable of sequential release by printing through the coaxial nozzle. They fabricated the construct by 3D-printing the outer Alg layer and an inner PLGA core. Both polymers were capable of differential release profiles of fluorescent dyes. They observed the sequential release of fluorescein. This retention was facilitated due to the delayed release of it from the PLGA core that should pass the Alg layer. While rhodamine B encapsulated in the Alg layer released more rapidly. The graph presented the release of both dyes during 150 h [150].

**Figure 10:**
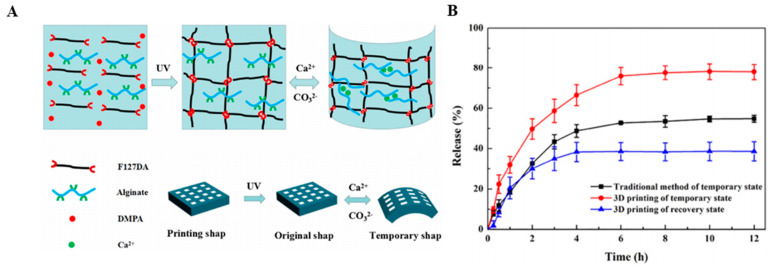
(**A**) The molecular mechanism for shape memory feature of printed hydrogels. (**B**) The hydrogels methotrexate (MTX) release profiles [151].

Improving mechanical strength by adding PLGA to Alg, lack of cytotoxicity, which was confirmed by incubating the scaffold with the human embryonic kidney (HEK293) cell line and bone marrow stromal stem cells (BMSC) is another positive point of this 3D construct. Previous properties made this bioink a suitable choice for the controlled differential release of drugs or proteins through such a delivery system, from treating cancer to regenerative medicine [150]. However, there are some situations where drugs should be released rapidly. Wang et al. [151], tried to make the shape memory hydrogel (SMHs) with Alg and pluronic F127 diacrylate macromer (F127DA) as a drug carrier through 3D printing technology as can be observed in Figure 10A [151]. Methotrexate (MTX) clinical anticancer drug was encapsulated in the hydrogel by the formation of the dual network structure. Results showed the rapid drug release, which was because the internal mesh structure increases the surface area ratio of the drug-loaded hydrogel, as shown in Figure 10B [151]. The performance of rapid drug release offers potential applications for local release anesthesia or hemostasis drugs in clinical operations. The F127DA UV cross-linking network mechanism for the gel structure provides mechanical support [151]. Based on this research, the hydrogel high recovery ratio in a short time, the low cytotoxicity and excellent biocompatibility of hydrogel may have applications in tissue engineering or drug delivery in clinical surgery [151]. To sum up, Alg has proved itself as an important drug delivery tool due to its versatile favorable characteristics. It is widely used in pharmaceutical research as a controlled-release polymer. Being biodegradable and biocompatible, Alg is made adaptable to different high-tech fabrication methods, such as 3D printing. In addition, 3D bioprinting gives the researcher many opportunities to engineer delivery systems that provide the preferred performance by tailoring the assembly process for given material composition. Considering this situation, it is inevitable that these methods will become routine in drug delivery and serve individual preferences by providing personalized drug delivery systems [151]. However, it is widely recognized that the tumor microenvironment performs an important function in regulating the action of numerous drugs on the tumor. Bioprinting has demonstrated a guarantee in manufacturing tumor versions with improved biological similarity over their 2D alternatives [152]. This is actually confirmed in a glioma model created employing glioma stem cell-laden Alg/Gel/fibrinogen bioink in Dai et al. [153] study. Their product revealed improved resistance to the drug temozolomide in comparison with a 2D culture product. Alg-based 3D printing highly customized products drug delivery systems are promising as they can overcome the drawbacks associated with conventional drug delivery. The tissue engineering and pharmaceutical applications of Alg printed constructs, the outstanding achievements of interesting studies in this field, and additives and printing methods are all summarized in Table 2.

## 6. Limitations, Advantages and Future Prospects of Alg 3D Printing

Additive manufacturing (AM) approaches offer essential progress in tissue engineering due to plenty of the parameters of the structure that regulate the physical, mechanical, and degradation properties of the scaffolds and are adaptable within relatively wide limits. Furthermore, they have the capability to print living cells [154]. 3D-bioprinted tissue constructs are appearing designed not just for transplantation but additionally for employment in drug discovery, evaluation of chemical, biological and toxicological agents, and introductory study. Therefore, the accomplishment of an adjusted ink and approach is essential in fabricating 3D printed constructs for drug delivery systems [155]. Regardless of the numerous benefits, 3D bioprinting poses many issues which ought to be resolved; these consist of: (1) recognition of biodegradable and biomimetic printable components that permit quick cell adhesion and growth; (2) the demand for vascularization at the single-cell stage; (3) complicated patterning of heterocellular tissues; and (4) preserving cell viability and long-term performance post-printing until eventually redesigning and reproduction is finished [1]. Algs with exceptional characteristics such as their gelling ability, low degree of toxicity, excellent availability, and cost-effectiveness are already used regarding the creation of bioinks. Alg hydrogels particularly offer a suitable marketplace, scaffolding, and providing a suitable matrix for cell loading. Shear-thinning characteristic, quick crosslinking capacity, and the possibility of viable printing cells create Alg among the most prosperous bioink. Algs are likewise accessible in a wide variety of MW and viscosities; alternatively, the removal procedure may directly affect the characteristics of the ultimate attained Alg [24]. Nevertheless, the Alg does not reach the structural features needed to perform as mechanical aid in HT engineering and exhibits negligible cellular attachment and sluggish degradation rate, along with low cell proliferation [24]. In this context, researchers are seeking out alternatives to these types of challenges, and they have identified various approaches; for instance, encapsulation of numerous growth factors (e.g., transforming growth factor; TGFα) to Alg-based structures might enhance the cell attachment [24]. A similar result was observed when Arg-Gly-Asp adhesion peptides were embedded into Alg bioink [22]. Selecting bioprinting approaches coupled with suitable bioinks is a crucial factor for the triumphant manufacturing of tissues, although numerous disadvantages ought to be addressed. Regarding Alg, there are two primary challenges connected with the bioprinting of Alg. First of all, it is challenging to print 3D cell-laden Alg scaffolds with entirely interconnected pores because of the issues in handling the gelation procedure. Next, the thickness of the structure which might be printed is restricted due to the fact Alg presented great interactions with water and negligible viscosity limiting thick 3D construct. Remarkably, considering that Alg is exceptionally soluble in aqueous media, distribution of Alg straightaway in CaCl_2_ media might deteriorate the structure [22]. The progression of perfect bioink is yet in development, and because of the considerable contributions from all over the world, it could be feasible to employ this system for industrial purposes in the near future. There is still a requirement to improve the ultimate properties attained with Alg-based components for their possible usage in 3D printing approaches for drug delivery systems. Aside from the bioinks, it is regarded that the progression of innovative bioprinters with outstanding resolution and lower prices could improve the potentials of this research field. The rise of bioinks and 3D bioprinting is appealing, resulting in the progression of innovative patient-certain tissue/organs and products in the potential future [29]. It is worth noting that we are still a great distance from organ printing. Even though recent deposition and manufacturing systems permit us to develop structures similar to tissue in their composition, the progression of entirely functioning tissue is a much more significant step [154,155,156,157,158,159,160,161,162,163,164,165,166,167,168,169,170,171,172,173,174,175,176,177].

## 7. Conclusions

Three-dimensional (3D) printing approaches for biological purposes attract great attention throughout the last ten years, and these approaches have started out new paths and directions in regenerative investigation. Despite the fact this approach is a reasonably early step of progression, 3D bioprinting tissue with this approach has presented great results. 3D printing approaches have the possibility to be extensively employed in tissue engineering (TE) fields, such as examination of antibacterial and biological agents, drug release, organ products, and some other biomedical purposes. These aims require obtaining adjusted bioinks. According to the author’s knowledge, alginate (Alg) is among the most widely used natural hydrogels bioinks that reveal distinctive characteristics including cytocompatibility, affordable price, various options of crosslinking, and compatibility with numerous approaches of printing. Nevertheless, Alg possesses a number of downsides, including insufficient structural stability; poor mechanical characteristics, and it points out low cell attachment than that of other natural polymers. To solve these issues, investigators used a combination of Alg along with other natural or synthetic polymers and encapsulated it with reinforcement agents (ceramics and carbon nanoparticles). We expect this review article will aid other investigators to enhance Alg-based bioinks via utilizing prior approaches described here, or to motivate novel bioink formulations for upcoming 3D bioprinting research. However, outstanding mechanical performance, suitable printability, and great cell response for 3D bioprinted tissue and organ constructs continue to be ambiguous. The foreseeable future is vibrant for the progression of human implantable 3D bioprinted Alg-based bioinks for regenerative medicine and biomedical applications.

## Figures and Tables

**Table 1:** Various biomedical fields of Alg.

Material	Fabrication Method	Crosslinking Type	Structure	Cells Viability and Attachments Support	Cell Type	Mechanical Results	Application	Reference
Alg/CPC	Extruding Alg-cell droplets into wells containing calcium chloride	Ionically with calcium chloride	Injectable hydrogel	Well hUCMSCs viability, osteo differentiation, and formation of bone minerals	hUCMSCs	Constructed mechanical properties were similar to cancellous bone. The flexural strength of CPC-chitosan-fiber-microbead ≈ 4 MPa	BTE	[44]
Alg/anti-BMP2 monoclonal antibodies	Extruding of Alg droplets through a syringe into calcium chloride solution	Ionically with calcium chloride	Microspheres	Well osteo differentiation (in vitro), new bone formation (in vivo)	MSC	-	BTE	[45]
Alg/RGD peptide	Reacting with peptide and cross linking	Ionically with CaSO_4_	Hydrogels	High osteoblast attachment and spreading	MC3T3-E1 osteoblast	-	BTE	[46]
Na-Alg	Gelation	Ionically with CaCl_2_	Gel	Proliferation of BMCs were faster on MVG gels than LVM	BMCs	Tensile strength for MVG ≈ 240 KPa and LVM ≈ 280 KPa	BTE	[47]
HAp/Alg/CS	In situ coprecipitation, freeze-drying	Ionically with CaCl_2_	Scaffolds	No cytotoxic and (in vitro), new bone generation (in vivo)	MG-63 cells	-	BTE	[48]
Alg/fibrin	Loading in a syringe which was equipped with device of bead generating	Ionically with calcium chloride	Microbeads	Highly expressions of bone markers such as ALP, OC, collagen I, and Runx2	hUCMSCs	-	BTE	[49]
Alg/CS/HAp	Distribution and freeze drying	Ionically with CaCl_2_	Scaffolds	High differentiation and mineralization	MC3T3-E1	Compressive strength = 0.68 MPa and elastic modulus = 13.35 MPa	BTE	[50]
Alg/CS/Gl/HAp	Simple foaming	Glutaraldehyde (for CS)	Scaffolds	Well Cell attachment, viability, proliferation	Osteoblast	-	BTE	[51]
Alg/CS/silica	Blending and freeze drying	CaCl_2_	Scaffolds	No significant toxicity	Osteoprogenitor, MG63, HOS	Compressive stress = 0.59 ± 0.405 Mpa, Young’s Modulus = 8.16 ± 0.567 MPa	BTE	[52]
Na-Alg	Mixing with syringe	CaSO_4_/CaCl_2_	Gel system	Chondrocytes viability for several weeks	Chondrocytes	Young’s Modulus ≈ 0.17 ± 0.01 MPa	CTTE	[53]
Alg	Using microfluidic device	Ionically with calcium chloride	Scaffolds	Nontoxic, well cells proliferation and maintaining cells phenotypes	Chondrocytes	-	CTTE	[54]
Alg/GL	Casting	Ionically with Ca^2+^	Film	Suitable cell proliferation, cell differentiation for same blend specimen	C_2_C1_2_ myoblasts	-	CTE	[55]
Alg/RGD peptide	Freeze drying	Ionically with calcium ions	Scaffolds	High cell adherence to the matrix, acceleration of cardiac tissue regeneration	Neonatal rat cardiac cells	-	CTE	[56]
Alg	Freeze drying	-	Scaffolds	Favorable cells viability, urea and albumin secretion	Hepatocyte	-	Liver TE	[57]
Na-Alg/CS	Spinning of Alg into a coagulation system	-	Hybrid fibers	Well fibroblast adhesion, Fibroblast produce collagen I fibers	Fibroblast	Tensile Strength > 200 MPa.	Ligament and tendon TE	[58]
Alg	Injection of oxidized Alg into a Teflon mold.	Ionically cross-linked with calcium ions	Hydrogels	Improvement of cartilage-like tissue formation (in vivo)	Chondrocyte	-	TE	[59]
Na-Alg/PEO	Electrospinning	Ionically with CaCl_2_	Core–shell nanofibers	Nontoxic	Fibroblasts cells	-	TE	[60]
Alg	Gelation and casting in a Teflon mold.	Ionically with Ca^2+^	Scaffolds	-	-	In high Ca content: compressive modulus ≈ 70 KPa and compressive strength ≈ 590 KPa	TE	[61]
AAlg/PNIPAAm	Mixing solutions, dialyzing and freeze drying	-	Injectable hydrogel	Nontoxic	hBMSCs	-	TE	[62]
Alg/CS/GRGDSP peptide/PEO	Electrospinning	Mixing and lyophilization	Nanofibers	Favorable cells attachment and proliferation	MC3T3	-	TE	[63]
Alg/lignin/CaCO_3_	Gelation with Pressurized carbon dioxide	CaCO_3_ (releasing Ca^2+^)	Aerogels	Non-cytotoxic, well cell adhesion	L929	Young’s Modulus = 1.36 ± 0.24 MPa	TE	[64]
Alg/PEO/GRGDSP	Electrospinning	Ionically with CaCl_2_	Nanofibers	Good adhesion, spread, and proliferation	HDF	-	TE	[65]
Alg/HNTs	Mixing of solutions and freeze drying	Ionically with calcium chloride	Scaffolds	Good cell attachment and proliferation	NIH3T3	-	TE	[66]
Alg/PLGA	Water/oil emulsion, lyophilization	Ionically with calcium chloride	Microspheres	Increase of NPCs expansion rate	NPCs	-	TE	[67]
Alg/TGF-1	Mixing and gelation	Ionically with calcium chloride	Hydrogel	Induction of odontoblast-like cell differentiation	Odontoblast-like cell	-	Repair human dental pulp	[68]
Alg/CPC	Directly injection into a fibrous structure in a Ca^2+^ containing bath	Ionically with Ca^2+^	Scaffolds	Favorable cells proliferation and osteo differentiation, new bone tissue formation and the defect closing (in vivo)	MSCs	-	Drug delivery and BTE	[69]
Alg	Reacting with glutaraldehyde + carboxylate moieties as stimuli responsive sensors	Glutaraldehyde	Stimuli-responsive hydroge	-	-	-	Drug delivery and TE	[70]
Na-Alg/isoniazid	Emulsification	Ionically with calcium chloride	Spherical microspheres	-	-	-	Oral sustained drug delivery	[71]
Alg/CS	Alg/chitosan solution was dripped via needle	Dual crosslinking with Ca^2+^ and SO_4_^2−^	Beads	-	-	-	Oral drug delivery	[72]
CAlg/CMC/5-FU	Ionic gelation, extruding solutions via syringe	Ionically with calcium chloride	Beads	Significant reduction of cells viability for all formulations after 48 h	Colon adenocarcinoma cells (HT-29)	-	Colon drug delivery	[73]
Alg/CS, AlgGO/MZ	Solutions dropped into calcium pantothenate and left at room temperature	-	Beads	-	-	-	Stomach drug delivery	[74]
Terbutaline sulfate and (BSA)-loaded Alg–poloxamer	Emulsification + external gelation	Ionically with CaCl_2_	Microparticle	-	-	-	Drug delivery to mucosal tissue	[75]
Alg/CS/5-FU and tegafur	Extruding of solutions through a syringe into calcium chloride solution	Ionically with calcium chloride	Microparticle	-	-	-	Drug delivery	[76]
Alg/N-Succinyl CS/nife- dipine	Ionic gelation	Ionically with calcium chloride	Beads	-	-	-	Drug delivery	[77]
Alg/CaCO_3_	Templating water-in-oil emulsion and subsequent in situ gelation	Ionically with Ca^2+^	Core-shell hydrogel beads	-	-	-	Drug delivery	[78]
Na-Alg/HPMC/CaGlu	Direct compression	Ionically with calcium ions	Matrices	-	-	-	Drug delivery	[79]
Alg/peptides derived from laminin and elastin	Using commercial calcium Alg dressing and cross linking with the peptide	Hybrid peptides derived from laminin and elastin	Fibrous dressings	Enhancement of NHDF proliferation (in vitro), significantly greater epithelialization and a larger volume of regenerated tissue (in vivo)	NHDF	-	Wound dressings	[80]
Alg/PVP/nano-silver	Mixing Al and PVP, heating and irradiation	Gamma irradiation	Hydrogel network	-	-	-	Wound dressings	[81]
Alg/CS	Gelation and lyophilization	Ionically with calcium chloride	Sponge	-	-	-	Wound dressings	[82]
Na-Alg/PC/GL/SIM	Solvent casting	-	Film	Nontoxic	HDF	Tensile strength SA–PC–SIM ≈ 3.32 ± 0.58, N/mm^2^ SA–GL–SIM ≈ 1.78 ± 0.02 N/mm^2^	Wound dressings	[83]
Na-Alg/ibuprofen)	Casting and pouring in petri dish	-	Hydrocolloid films	The bilayer films treated wound faster than single layer SA films (in vivo)	-	Tensile strength ≈ 27.22 ± 0.95 MPa	Wound dressings	[84]
Oxidized Alg/GL	Via syringe fibrin glue applicator	AlgDA	Hydrogel	Acceleration of epithelialization	-	-	Wound dressings	[85]
Na-Alg/PVA/ZnO	Electrospinning	Glutaraldehyde, CaCl_2_	Nanofibers	Less toxic in 0.5 and 1% ZnO	L929	-	Wound dressings	[86]
Na-Alg	Syringe pumps infused solutions into the microfluidic device	Ionically with Ca^2+^	Microbeads	Cells viability enhanced from 19.3% to 74.3% with the increase of CaCO_3_ concentration from 1.14 to 9.10 mg∙mL^−1^	Mammalian cells (Jurkat)	-	Cell Encapsulation	[87]
Alg	Microfluidic technique	Ionically with Ca^2+^	Hydrogel microparticles	Cells viability over 98%	Yeast cells	-	Cell encapsulation	[88]
Alg	Mixing and pouring molds and using UV	Photoinitiator VA-086	Hydrogel scaffolds	Noncytotoxic	Chondrocyte	10–20 kPa modulus range	Cell encapsulation	[89]

**Table 2:** Various 3D printed Alg based structures in biomedical applications.

Material	Printing Method	Cell Type	Target Tissue	Results	Reference
Alg/gelatin scaffolds with nano apatite coating	Bioscaffolder 3.1, Gesim	Rat bone marrow stem cells	Bone	Alg/gelatin scaffolds with nano apatite coating had 2-fold higher Young’s Modulus compared with the scaffolds without coating, stimulated the proliferation and osteogenic differentiation of rat bone marrow stem cells	[26]
Na-Alg and calcium phosphate	A custom-designed 3D printer	-	Bone	The compressive strength of composite materials increased with Alg concentration from 0.45 Mpa up to about 1.0 MPa	[27]
Bioactive glass/Alg	Bioscaffolder 2.1 platform (GeSiM) (extrusion based)	BMSCs	Bone	Shrinkage ratios decreased by half, 13–93 BG incorporation improved the compressive strength and modulus of the SA scaffold, achieving highest values of 16.74 ± 1.78 MPa and 79.49 ± 7.38 MPa	[28]
Na-Alg/gelatin/nano hydroxyapatite/hASCs	3D Bioplotter	Human adipose-derived stem cells (hASCs)	Bone	New formation of bone in existence of HA was higher than in the Alg/gelatin	[98]
Alg and alkaline phosphatase (ALP)	3D Bioplotter	MG-63 osteoblast-like cells	Bone	Mineralization was observed in all scaffolds containing ALP	[101]
Alg/Alg-sulfate/bone morphogenetic protein 2	Custom-made 3D bioprinting system (extrusion based)	MC3T3-E1 osteoblasts	Bone	Improve retention of bone morphogenetic protein 2, promote osteoblastic proliferation and differentiation	[102]
Alg/graphene oxide	Custom-made 3D bioprinting system	Mesenchymal stem cells (MSCs)	Bone	Improve printability, structural stability, and osteogenic activities	[103]
Alg/bone formation peptide-1(BFP1)	3D printer	human adipose-derived stem cells (hADSCs)	Bone	Good cell viability and proliferation, enhancement bone regeneration activities	[104]
Alg/Hydroxyapatite/Atsttrin	3D bioprinter	C3H cells	Bone	Promote bone defect repair	[105]
Alg/Nanocellulose	Stereolithography (for ear painting)	Human chondrocytes, L929	Cartilage	Excellent printability at room temperature, high shape fidelity	[107]
Alg Sulfate/Nanocellulose	BioFactory, regenHU	Bovine chondrocytes	Cartilage	Very good printability, promote cell spreading, proliferation, and collagen II synthesis	[108]
Na-Alg/Collagen (Col) or agarose (AG)	A 3D–Bioplotter	Chondrocytes	Cartilage	The mechanical strength was improved in both SA/COL and SA/AG groups compared to SA alone, incorporation of COL could distinctly enhance cell adhesion, proliferation and the expression of cartilage specific genes such as Acan, Col2al and Sox9	[109]
Alg/PCL	Additive manufacturing (AM) with a multihead deposition system (MHDS)	Chondrocyte	Cartilage	PCL–Alg gels containing transforming growth factor-b (TGFb) showed higher ECM formation. Enhancement of cartilage tissue and collagen II	[110]
				formation in the PCL–Alg gel (+TGFb) hybrid scaffold	
Alg/gelatin	Dual-syringe Fab@Home bioprinter	Aortic root sinus smooth muscle cells (SMC) and aortic valve leaflet interstitial cells (VIC)	Cardiac	High cells viability, anatomically complex, heterogeneously encapsulated aortic valve hydrogel conduits could fabricate with 3D bioprinting	[115]
Calcium Alg	Novel 3D bioprinting method by using a coaxial nozzle	L929	Vascular	High-strength structures, good cell viability	[118]
Na-Alg	Coaxial deposition system	Human umbilical vein smooth muscle cells (HUVSMCs)	Vascular	Good proliferation, deposition of smooth muscle matrix and collagen	[119]
Na-Alg	Inkjet Printing	Fibroblast (3T3 cell)	Vascular	Cell viability of printed cellular tubes has been found above 82%	[120]
Na-Alg	Inkjet Printing	Mouse fibroblast	Vascular	Fibroblast cell viability of printed cellular tubes was found to be above 90%, successfully print of vascular-like structures	[121]
Gelatin methacryloyl (GelMA), sodium Alg, and 4-arm poly(ethylene glycol)-tetra-acrylate (PEGTA)	Multilayered coaxial extrusion systems	Endothelial and stem cells	Vascular	Favorable physicochemical characteristics, support of the proliferation and early maturation of vascular cells, bioprinting of complex 3D vasculature	[122]
Alg	Extrusion-based	HepG2	Liver	Enhancement of cell viability, control of cellular level differentiation, control of tissue level function	[125]
Alg/Hyaluronic Acid (HA)	Extrusion-based	Schwann	Nerve	Scaffolds created from this bioprinting process can support the performance of encapsulated Schwann cell viability, proliferation and cellular protein expression	[127]
Alg/Agarose/carboxymethyl-chitosan (CMC)	Extrusion-based	Human neural stem cells	Nerve	Formation of stable cells encapsulated 3D structures	[128]
Pluronic/Alg	Extrusion-based	Myoblast	Muscle	Improved viability of cells compared to the normal 2D cultures	[129]
Alg/Gelatin	Extrusion-based	A549	Lung	The first attempt to generate 3D bioprinted humanized models for infection studies	[130]
Gelatin/Alg	Bio-extrusion	Primary human skin cells	Skin	Cells spreading induced a rapid differentiation of the dermis, leading to a stiff tissue on which epidermis can be rapidly seeded	[131]
Gelatin/Alg	Extrusion free-forming system	Human skin fibroblasts (HSFs)	Skin	Dual crosslinking process can make dermal substitute scaffolds with physicochemical properties that match with human skin tissue	[132]
Gelatin/Alg	Extrusion-based	-	Skin	Enhanced the detachment of the crusts, decreased the agglomeration of keratinized substances	[140]
Alg/PLGA	Extruded type	-	Skin	Promoted cell adhesion and proliferation promote neovascularization and collagen I/III deposition	[141]
Alg/PLGA	Coaxial extrusion-based system	Human embryonic kidney (HEK293)	Kidney	Sequential release and retention of drug lack of cytotoxicity improving mechanical strength	[150]
Alg/pluronic F127 diacrylate macromer (F127DA)	Extruded type	3T3 cells	Drug delivery in vascular tissue	Rapid drug release high recovery ratio in a short time the low cytotoxicity	[151]

## Abbreviations

AAlg	Aminated alginate
AlgDA	Alginate dialdehyde
AG	Agarose
Alg	Alginate
AlgGO	Alginate gel bead containing vegetable oil
ALP	Alkaline phosphatase
AM	Additive manufacturing
BFP1	Bone formation peptide-1
BLM	Bilayer membrane
BMCs	Bone marrow cells
BSA	Bovine serum albumin
BTE	Bone tissue engineering
CAlg	Calcium alginate
CaGlu	Calcium gluconate
CMC	Carboxymethyl cellulose
COL	Collagen
CP	Calcium phosphate
CPC	Calcium phosphate cement
CS	Chitosan
CTE	Cardiac tissue engineering
CTTE	Cartilage tissue engineering
ECM	Extracellular matrix
FDM	Fused deposition modeling
GelMA	Gelatin methacryloyl
GL	Gelatin
GO	Graphene oxide
GRGDSP	Glycine–arginine–glycine–aspartic acid–serine–proline
HA	Hyaluronic acid
HAp	Hydroxyapatite
hBMSCs	Human bone mesenchymal stem cells
HDF	Primary human dermal fibroblast cells
HEK	Human embryonic kidney
HOS	Human osteosarcoma cell lines
HNTs	Halloysite nanotube
HPMC	Hydroxypropylmethylcellulose
HSFs	Human skin fibroblasts
HUCMSCs	Human umbilical cord mesenchymal stem cells
HUVSMCs	Human umbilical vein smooth muscle cells
MSCs	Mesenchymal stem cells
MTX	Methotrexate
MZ	Metronidazole
NFC	Nano fibrillated cellulose
NHDF	Normal human dermal fibroblasts
NPCs	Neural progenitor cells
PC	Pectin
PEO	Poly(ethylene oxide)
PEGTA	Poly (ethylene glycol)-tetra-acrylate
PGRN	Progranulin
PLGA	Poly (lactide-co-glycolide)
PNIPAAm	Poly (N-isopropylacrylamide)
PVA	Poly (vinyl alcohol)
PVP	Polyvinyl pyrrolidone
rGO	reduced graphene oxide
Na-Alg	Sodium alginate
SIM	Simvastatin
SLS	Selective laser sintering
SMC	Smooth muscle cells
SMHs	Shape memory hydrogel
5-FU	5-fluorouracil
TE	Tissue engineering
TGF-1	Transforming growth factor 1
TIJ	Thermal inkjet
(TNF)-α	Tumor necrosis factor
UV light	Ultraviolet light
VIC	Valve leaflet interstitial cells
3D	Three-dimensional
